# Performance evaluation of GPT-4o on South Korean national exams for building mechanical equipment maintenance

**DOI:** 10.1038/s41598-025-16118-x

**Published:** 2025-08-19

**Authors:** Haneul Choi, Jehyun Lee, Jonghun Kim

**Affiliations:** 1 Energy ICT Research Department, Korea Institute of Energy Research, Daejeon, 34129 South Korea; 2 Energy AI & Computational Science Laboratory, Korea Institute of Energy Research, Daejeon, 34129 South Korea

**Keywords:** GPT, Large language model, Mechanical equipment maintenance manager, Building operator, Engineer energy management, Engineer air-conditioning refrigerating machinery, Civil engineering, Mechanical engineering

## Abstract

This study evaluates the applicability of large language models (LLMs) in mechanical equipment maintenance in buildings by assessing GPT-4o’s performance on two national certification exams in South Korea: Engineer Energy Management (EEM) and Engineer Air-Conditioning Refrigerating Machinery (EACRM). GPT-4o achieved average scores of 80.6 and 81.25 on the EEM and EACRM exams, respectively, passing all five attempts. The model performed well on both non-calculation and calculation problems and demonstrated high consistency, with an average response consistency of 97%. Despite these strengths, three key limitations were identified: weak advanced reasoning, difficulty in solving legal questions, and poor interpretation of scientific figures. Experimental results indicate that advanced reasoning can be improved using reasoning-optimized models, while legal question accuracy can be significantly enhanced with retrieval-augmented generation (RAG). However, figure interpretation remains dependent on advancements in visual recognition capabilities. These findings suggest that GPT-4o possesses foundational knowledge applicable to mechanical equipment maintenance in buildings but also highlight the need to address certain limitations for practical implementation. This study provides a foundation for future research on integrating LLMs into industrial applications, such as maintenance management software, to enhance maintenance efficiency and address workforce shortages.

## Introduction

Over the past two decades, research has highlighted a substantial increase in global energy consumption, primarily driven by rapid urbanization, population growth, economic expansion, and the rising demands and expectations of building occupants and owners^1^. According to the latest report by the International Energy Agency (IEA), global final energy consumption reached 445 exajoules (EJ) in 2023, with the building sector accounting for 125 EJ, representing approximately 28% of total consumption^2^. Among the various energy-consuming services in buildings, Heating, Ventilation, and Air Conditioning (HVAC) systems constitute the most energy-intensive category, contributing approximately 38% of total building energy consumption globally. The second-largest contributor is Domestic Hot Water (DHW), which accounts for approximately 13%^1^. These two services, collectively classified as mechanical equipment, account for nearly 50% of total building energy consumption, underscoring the critical role of efficient mechanical equipment management in mitigating global energy consumption.

South Korea also experiences significant energy expenditures on mechanical equipment. Annually, approximately USD 19.2 billion is allocated to energy costs for mechanical equipment, representing approximately 71% of total energy consumption in domestic buildings^3^. Reflecting global trends, the proportion of energy consumption attributed to mechanical equipment in South Korean buildings remains notably high. In response to this challenge, the Mechanical Equipment Act^4^ was enacted in 2018 and came into effect in 2020. The South Korean government introduced this legislation to enhance the efficiency of mechanical equipment management and maintenance in buildings, aiming to reduce energy costs by 10% annually. This reduction is projected to yield cost savings of approximately USD 1.92 billion per year while contributing to national energy efficiency and greenhouse gas emissions reduction efforts.

A key provision of the Mechanical Equipment Act is the mandatory appointment of a mechanical equipment maintenance manager in designated buildings. These managers are certified professionals holding nationally recognized qualifications and are responsible for ensuring occupant comfort while optimizing energy consumption through the inspection, management, and operation of mechanical equipment. According to the Enforcement Decree of the Mechanical Equipment Act^5^buildings with a total floor area exceeding 10,000 m² or residential complexes with more than 500 units must appoint a designated number of maintenance managers based on the building size. For instance, buildings with a total floor area exceeding 60,000 m² are required to employ one principal maintenance manager and one assistant maintenance manager, while buildings with a total floor area between 10,000 m² and 15,000 m² are required to employ one principal maintenance manager. Currently, more than 40,000 buildings in South Korea fall under this regulatory requirement.

Despite these regulatory efforts, the field of mechanical equipment maintenance faces two critical workforce challenges. First, the supply of qualified professionals is insufficient relative to the number of buildings subject to regulatory requirements. Many building owners and operators express concerns over the financial burden associated with appointing maintenance managers, with an estimated annual cost of USD 38,000 per manager. Moreover, suburban and rural areas experience acute labor shortages. Second, the workforce in this field is aging, with a disproportionate number of older professionals. As of August 2024, data from the Korea Mechanical Construction Contractors Association^6^ indicate that 91% of mechanical equipment maintenance managers are aged 40 years or older. Consequently, while the implementation of the Mechanical Equipment Act has expanded the scope and importance of mechanical equipment maintenance, the sector continues to face significant labor shortages.

To address these workforce challenges, recent research and industry initiatives have explored the potential of artificial intelligence (AI) and robotics as supplementary solutions^7–9^. In particular, the application of Large Language Models (LLMs) such as GPT^10^ has garnered increasing attention. LLMs are proficient in comprehending complex technical documents and extracting pertinent information, and they have been successfully implemented across various industries to assist human professionals. For example, Amazon has leveraged AI-powered chatbots based on LLMs to automate routine inquiries, reducing the workload of customer service representatives and enabling 24-hour support^11^. Similarly, NVIDIA and Hippocratic AI have announced plans to develop an LLM-based virtual nurse capable of providing human-level video consultations to mitigate the nursing workforce shortage in the United States^12^. Empirical studies have reported measurable productivity improvements attributable to LLMs. For instance, developers utilizing GitHub Copilot have been found to complete programming tasks 56% faster^13^while customer service agents using AI-based conversational assistants have demonstrated an average productivity increase of 14%^14^.

Given these advancements, an important question arises: Can LLMs be effectively utilized in the field of mechanical equipment maintenance? Specifically, could LLMs support tasks such as interpreting technical manuals, analyzing operational data, and assisting maintenance managers? A fundamental approach to evaluating the applicability of LLMs in a new domain is to assess their domain-specific knowledge. As summarized in Table 1, previous studies have demonstrated LLM proficiency across diverse disciplines. For example, healthcare is one of the most extensively studied domains in LLM knowledge evaluation, and LLMs have achieved high scores on various medical certification exams worldwide^15–19^. In the legal domain, GPT-4 ranked in the top 10% of examinees on the U.S. Bar Exam with a 90% accuracy rate^20^. LLMs have also demonstrated notable success in domain-specific assessments in fields such as agriculture^21^ and engineering^22–24^. More recently, a study reported that various LLMs, including GPT, possess a solid understanding of HVAC design, highlighting their growing potential in technical fields^24^.

**Table 1:** Research reviews of LLM performance across domains.

Domain	Exam type	LLM used	Performance	Ref.
Healthcare	Taiwan Internal Medicine Specialty Exam	GPT-4o, Claude 3.5 Sonnet, Gemini Advanced	Average accuracy of 78.88%	^15^
Healthcare	Graduate Biomedical Science Exams	GPT-4	Exceeded student average in 7/9 exams	^16^
Healthcare	Chinese Nursing Licensing Exam	Custom GPT, GPT-4	Up to 90+% accuracy	^17^
Healthcare	Registered Dietitian Exam	GPT-4o, Claude 3.5 Sonnet, Gemini 1.5 Pro	Up to 88+% accuracy	^18^
Healthcare	Turkish Dental Specialization Exam	GPT-4, Gemini Advanced	Maximum accuracy of 83.3%	^19^
Legal	US Bar Exam	GPT-4	Top 10% score	^20^
Agriculture	Certified Crop Adviser Exam	GPT-4, GPT-3.5, Llama 2	Maximum accuracy of 93%	^21^
Engineering	Fundamentals of Engineering Environmental Exam	GPT-4, GPT-3.5	Maximum accuracy of 75.37%	^22^
Engineering	Brazil ENADE Computer Science Exam	GPT-4 Vision	Top 10% score	^23^
Engineering	ASHRAE Certified HVAC Designer Exam	GPT-4, GPT-3.5, LLaMA	74–78 points	^24^

**Fig. 1:**
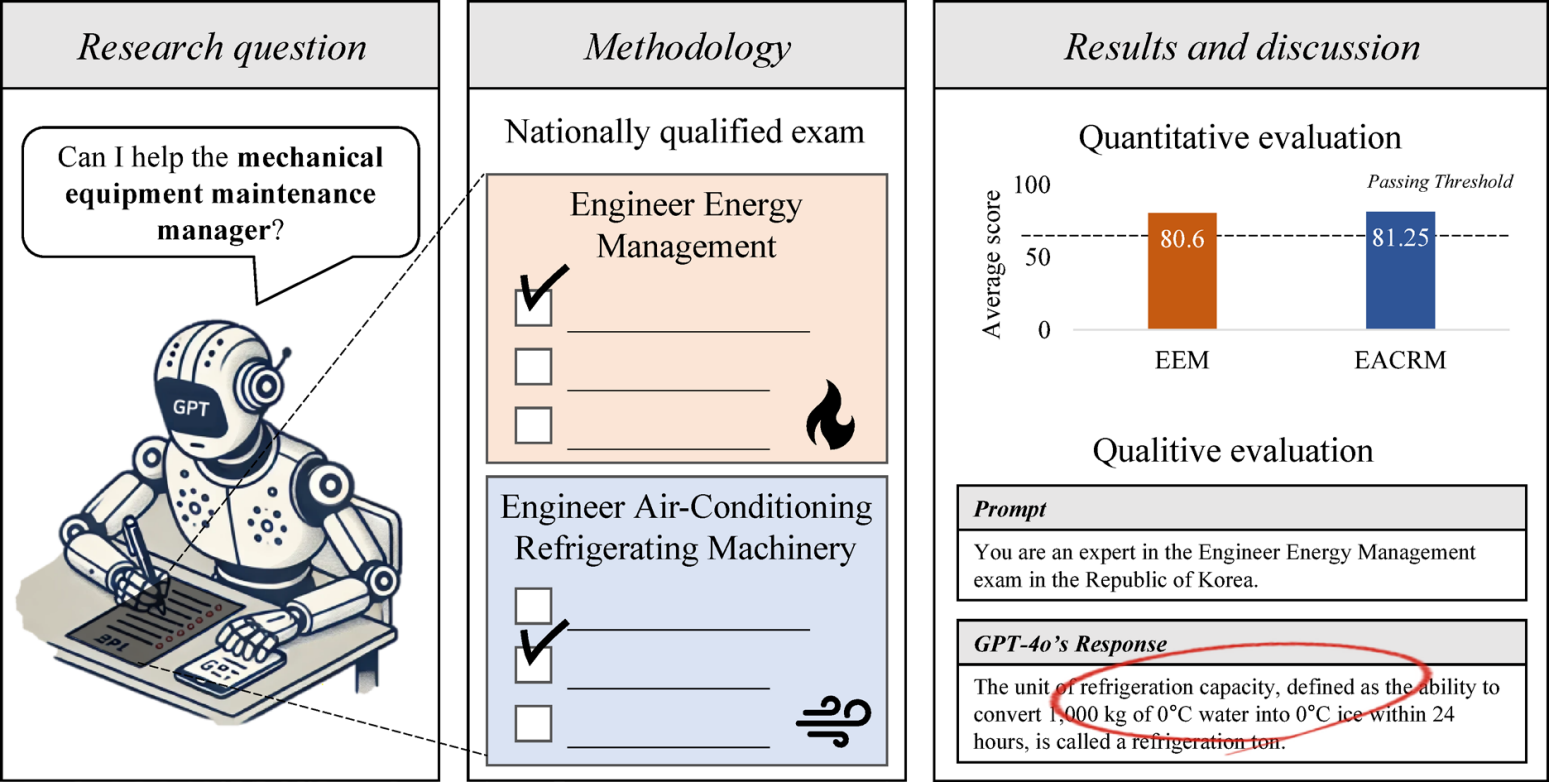
Research framework.

In this context, the present study aims to evaluate the applicability of LLMs in the domain of mechanical equipment maintenance in South Korea. Specifically, we investigate whether GPT, a representative LLM, possesses the requisite knowledge for mechanical equipment maintenance tasks. Given that certification as a mechanical equipment maintenance manager requires passing a national technical qualification examination, we assess GPT’s performance on the relevant exam questions. If GPT successfully passes the examination, it would provide evidence that the model possesses the foundational knowledge required for this professional role. Beyond simply determining whether GPT can pass the exam, this study also conducts a detailed analysis of its problem-solving process to identify strengths and limitations in its application to mechanical equipment maintenance. The research framework is illustrated in Fig. 1.

To the best of our knowledge, this is the first study to systematically evaluate the performance of LLMs on domain-specific national certification exams related to building mechanical equipment maintenance. Unlike prior work that has primarily focused on general language understanding or broad technical applications, this study uniquely explores how LLMs perform in highly specialized certification exams that directly reflect real-world engineering knowledge requirements. Although certification exams analyzed in this study are specific to South Korea, the engineering knowledge it assesses is largely applicable beyond national boundaries. The findings of this study can be utilized to develop practical methods for LLMs to assist mechanical equipment maintenance managers. If LLMs are integrated into maintenance tasks, they have the potential to address workforce shortages in the short term while enhancing maintenance efficiency in the long term, ultimately contributing to building energy savings.

## Methods

### Examinee

This study evaluated GPT-4o, released in May 2024, as the examinee. GPT is a LLM developed by OpenAI, capable of performing various tasks such as text generation, conversational responses, translation, document summarization, and code generation^10^. Since the release of GPT-1 in 2018, the model has undergone continuous development, with GPT-4 currently in use^20^. GPT-4o (Omni), introduced in May 2024, is a multimodal model capable of understanding and processing text, images, and voice.

### Exam questions

To assess GPT-4o’s knowledge level in mechanical equipment maintenance, this study used written test questions from two national technical qualification exams: Engineer Energy Management (EEM) and Engineer Air-Conditioning Refrigerating Machinery (EACRM). These two qualifications are the most commonly held by mechanical equipment maintenance managers, together accounting for approximately 40% of certified professionals. They are widely recognized as representative certifications in this field.

The EEM exam evaluates expertise in heating systems, including boilers and related equipment^25^. The written exam consists of five subjects: combustion engineering, thermodynamics, measurement methods, thermal equipment materials and related laws, and thermal equipment design. Each subject contains 20 single-choice questions, totaling 100 questions. The passing criteria require a minimum score of 40 points per subject and an average of 60 points across all subjects. The exam is conducted three times a year, and the past questions used in this study were from the second exam in 2022, which were publicly available online.

The EACRM exam focuses on refrigeration and air-conditioning systems, requiring knowledge of refrigeration and air-conditioning equipment^26^. The written exam consists of four subjects: energy management, air-conditioning and refrigeration design, commissioning and safety management, and maintenance and construction management. Each subject includes 20 single-choice questions, totaling 80 questions. The question format and passing criteria are identical to those of the EEM exam. Like the EEM exam, the EACRM exam is administered three times a year, and this study used past questions from the third exam in 2023.

Exam questions can be broadly classified into two types: non-calculation problems and calculation problems. Calculation problems typically require applying mechanical formulas or performing unit conversions, whereas non-calculation problems assess theoretical knowledge. In both exams, the ratio of non-calculation to calculation problems is approximately three to one. Most test questions are presented in text form, although some contain images. Since GPT-4o is a multimodal model capable of processing both text and images, image-based questions were not excluded. In rare cases where the accompanying text provided sufficient information to understand the question independently, the image was omitted and only the text was used. For all other image-based questions, the image path was specified to allow GPT-4o to access and interpret the visual content. Additionally, chemical formulas and mathematical equations were manually converted into ASCII-compatible representations to ensure accurate interpretation by GPT-4o. For example, “H₂O” was transcribed as “H2O”, and the equation “Q = m·c·ΔT” was expressed as “Q = mcΔT” using standard keyboard characters. All conversions were carried out carefully to preserve the semantic fidelity of the original expressions.

### Prompt design

**Fig. 2:**
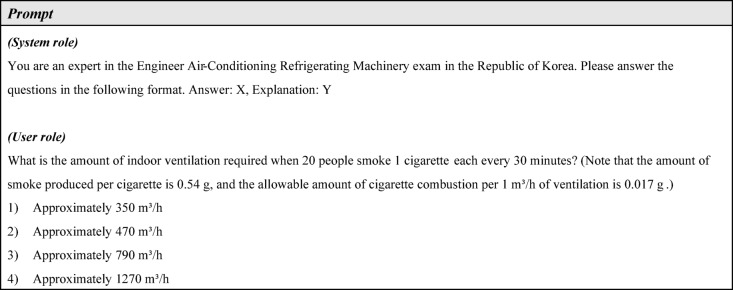
An example prompt used in the exam.

Prompts are instructions provided to GPT to guide its responses. In this study, prompts were structured into two roles: system role and user role. The system role defines GPT’s role as an examinee and specifies the required answer format. In this study, GPT-4o was assigned the role of an expert in the EEM or EACRM exam and was instructed to provide answers and explanations in a predefined format. Recent advancements in prompt engineering, which involve crafting sophisticated instructions for LLMs, have been shown to significantly enhance response accuracy^27,28^. However, to minimize the influence of prompt engineering, this study used basic and straightforward instructions to evaluate GPT-4o’s intrinsic knowledge. Meanwhile, the user role provided the exam questions and answer choices in their original format. An example of the prompts used in this study is illustrated in Fig. 2.

### Exam procedure

The test was conducted five times to account for the probabilistic nature of GPT’s responses. To ensure independent answers, GPT was prevented from referencing previous prompts during the testing process. This was achieved by utilizing the OpenAI API and custom code, rather than the ChatGPT interface. Python was used as the programming language for implementing the test procedure and processing the results.

One of the key adjustable parameters when using the OpenAI API is the temperature setting, which controls the randomness or creativity of the model’s responses. The temperature value ranges from 0 to 2, where a lower temperature (e.g., 0.2) produces more consistent and deterministic answers, while a higher temperature (e.g., 1.0) results in more varied and creative responses^29^. Previous studies have used different temperature values, including 0^22^, 0.3^20^, and 1.0^24^, depending on the nature of the task. Given the structured and objective nature of the test—specifically, its single-choice format that requires selecting one correct answer from four options rather than generating diverse responses—the temperature was set to 0.2 to ensure deterministic outputs and consistent evaluation across trials.

### Evaluation metrics

#### Subject scores and overall scores

The scores for each subject were calculated based on the scoring criteria for the EEM^25^ and EACRM^26^ exams. The EEM exam consists of five subjects, while the EACRM exam consists of four subjects. In both exams, the maximum score for each subject is 100 points. Since each subject contains 20 questions, each question is worth 5 points. If the score for any subject falls below 40 points, the exam is considered failed. The overall score is calculated as the average of the scores across all subjects. Since the full score for each subject is 100, the full score for the overall exam is also 100. If the overall score is below 60, the exam is considered failed.

#### Accuracy

The accuracy for each problem type (i.e., non-calculation problems and calculation problems) was calculated as the average accuracy of the problems within that type. The accuracy for each problem was determined using the following equation:

$$\:{Accuracy}_{i}=\frac{{Q}_{C,\:i}}{{Q}_{T}}\times\:100\left(\%\right)$$

Here, $\:{Q}_{c,\:i}$ represents the number of correct answers for the $\:i$-th problem, and $\:{Q}_{T}$ is the total number of tests (five in this study).

#### Consistency

Although the temperature, a hyperparameter of the model, is set to a low value, the correct answer may still vary across tests due to the probabilistic nature of GPT’s responses. If the answers are highly inconsistent, GPT cannot be reliably trusted to solve the problem. Therefore, this study evaluated the consistency of its answers. The consistency for each problem was calculated using the following equation:

$$\:{Consistency}_{i}=\frac{{C}_{i}}{{Q}_{T}}\times\:100\left(\%\right)$$

Here, $\:{C}_{i}$ represents the maximum number of identical answers given for the $\:i$-th problem across the five test iterations. If GPT selects the same answer for a given problem multiple times, those answers are considered identical.

#### Error distribution

The error distribution metric quantifies the distribution of error types found in GPT’s responses. It provides a structured view of the types of mistakes the model tends to make by measuring the relative proportion of each predefined error category. Based on prior research^30^errors were categorized into three types: reasoning error, knowledge error, and context error. A reasoning error refers to failures in the problem-solving process, such as incorrect formulation of equations or logical missteps. A knowledge error occurs when the model provides inaccurate information based on internal knowledge, such as engineering facts or legal concepts. A context error is defined as a failure to fully interpret or utilize the given input, such as omitting problem conditions or misinterpreting elements of a diagram. The percentage of each error type was calculated by dividing the number of instances for each category by the total number of errors across all responses.

## Results and discussions

### Quantitative evaluation

#### Subject scores and overall scores

**Fig. 3:**
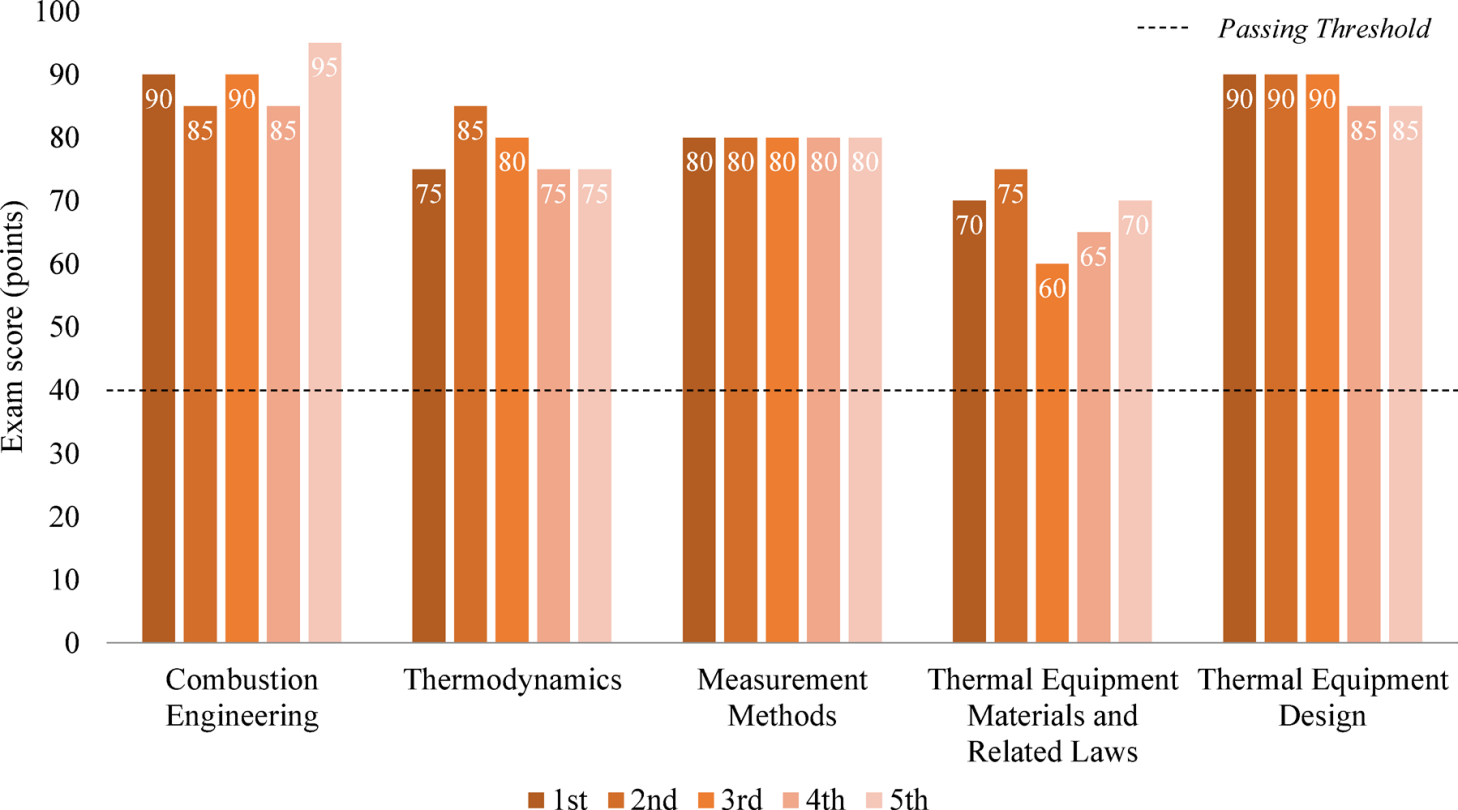
Subject scores for the engineer energy management exam

The average subject scores for the EEM exam across five test attempts are shown in Fig. 3. The scores were as follows: Combustion Engineering, 89 points; Thermodynamics, 78 points; Measurement Methods, 80 points; Thermal Equipment Materials and Related Laws, 68 points; and Thermal Equipment Design, 88 points. The highest score was recorded in Combustion Engineering, while the lowest score was in Thermal Equipment Materials and Related Laws. An examinee fails the exam if they score below 40 points in any subject; however, GPT scored at least 20 points above this threshold in all subjects.

**Fig. 4:**
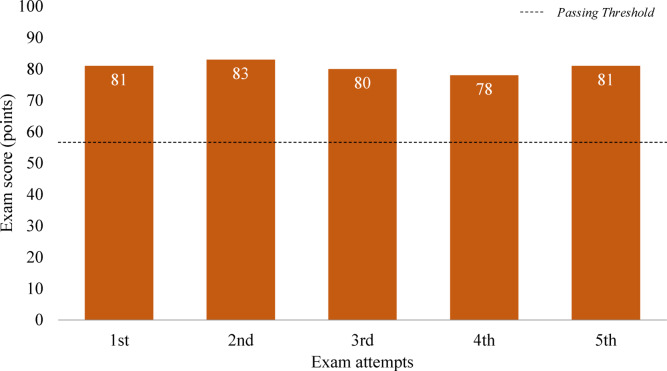
Overall score for the engineer energy management exam.

The average overall score across five attempts was 80.6 points (with a standard deviation (SD) of 1.82), as shown in Fig. 4. Across the five test attempts, the minimum score was 78 points, and the maximum score was 83 points. This score significantly exceeds the passing standard of 60 points. Overall, GPT-4o passed all five attempts, meeting both passing criteria—a minimum of 40 points per subject and an overall average of at least 60 points—achieving a 100% passing rate. This result is noteworthy, considering that the actual human passing rate for this exam is only 29%.

**Fig. 5:**
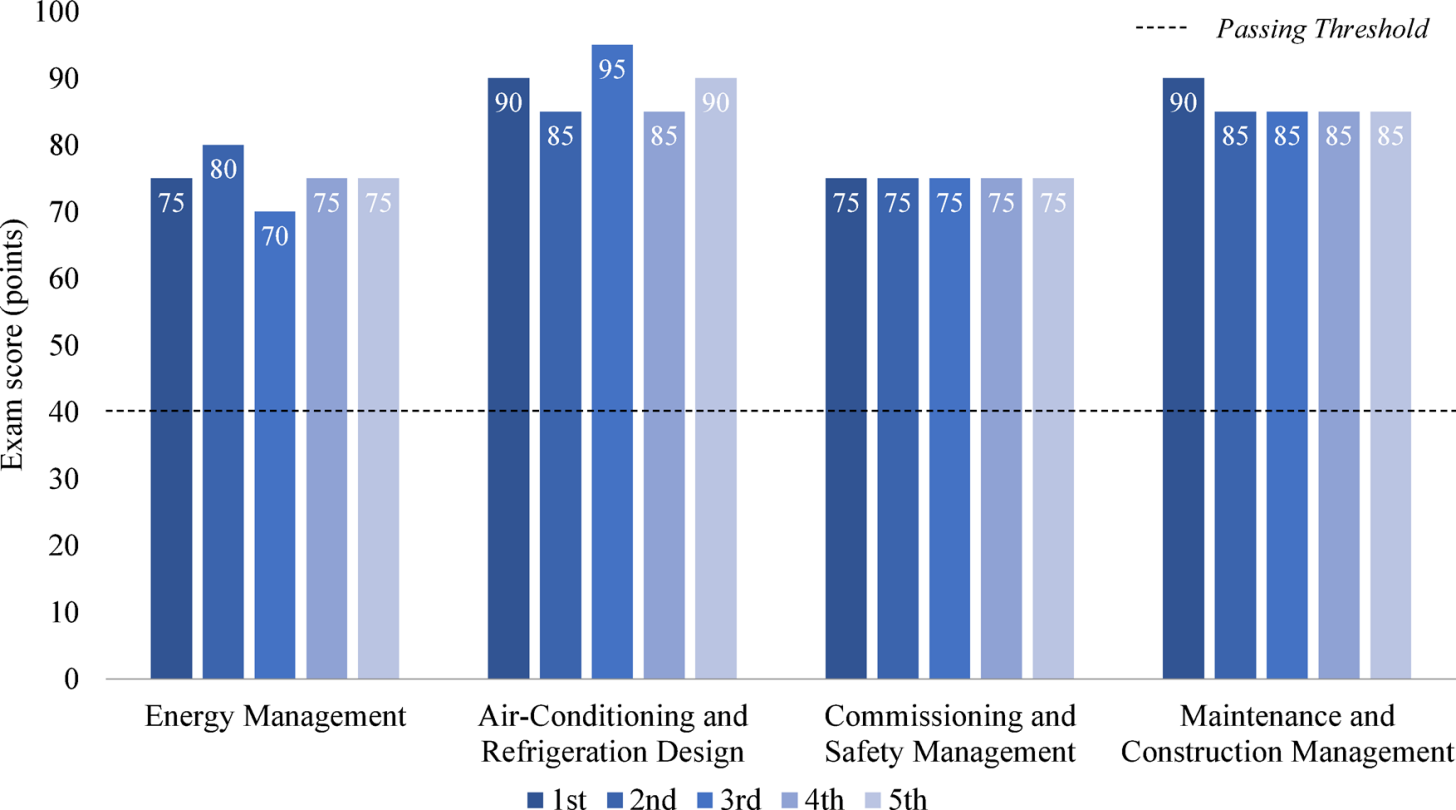
Subject scores for the engineer air-conditioning refrigerating machinery exam.

The average subject scores for the EACRM exam across five attempts are shown in Fig. 5. GPT-4o scored 75 points in Energy Management, 89 points in Air-Conditioning and Refrigeration Design, 75 points in Commissioning and Safety Management, and 86 points in Maintenance and Construction Management. The highest score was recorded in Air-Conditioning and Refrigeration Design, while the lowest scores were in Energy Management and Commissioning and Safety Management. An examinee fails the exam if they score less than 40 points in any subject; however, GPT-4o scored at least 20 points above this threshold in all subjects.

**Fig. 6:**
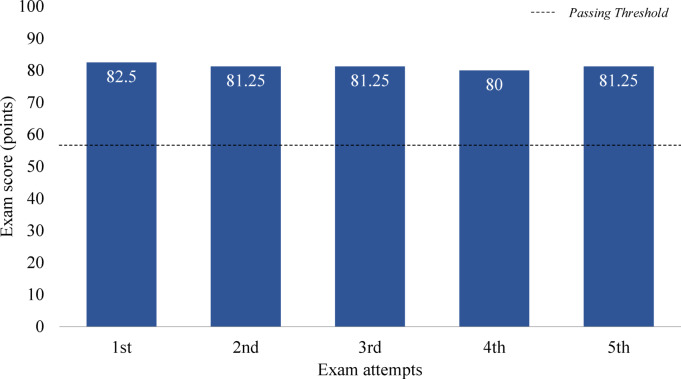
Overall score for the engineer air-conditioning refrigerating machinery exam.

The average overall score across five attempts was 81.25 points (SD = 0.88), with a minimum score of 80 points and a maximum score of 82.15 points, as shown in Fig. 6. This result is slightly higher than that of the EEM exam and significantly exceeds the overall passing standard of 60 points. Similar to the EEM exam, GPT-4o satisfied both passing criteria—a minimum of 40 points per subject and an overall score of at least 60 points—in all five attempts, achieving a 100% passing rate. Given that the actual human passing rate for this exam is only 31%, this result is also noteworthy.

In summary, GPT-4o achieved a high average overall score of approximately 80 points in both the EEM and EACRM exams, passing all five attempts with a 100% success rate. These results indicate that GPT-4o possesses fundamental knowledge of mechanical equipment maintenance and has the potential to support mechanical equipment maintenance tasks in the future.

#### The accuracy for each problem type

**Fig. 7:**
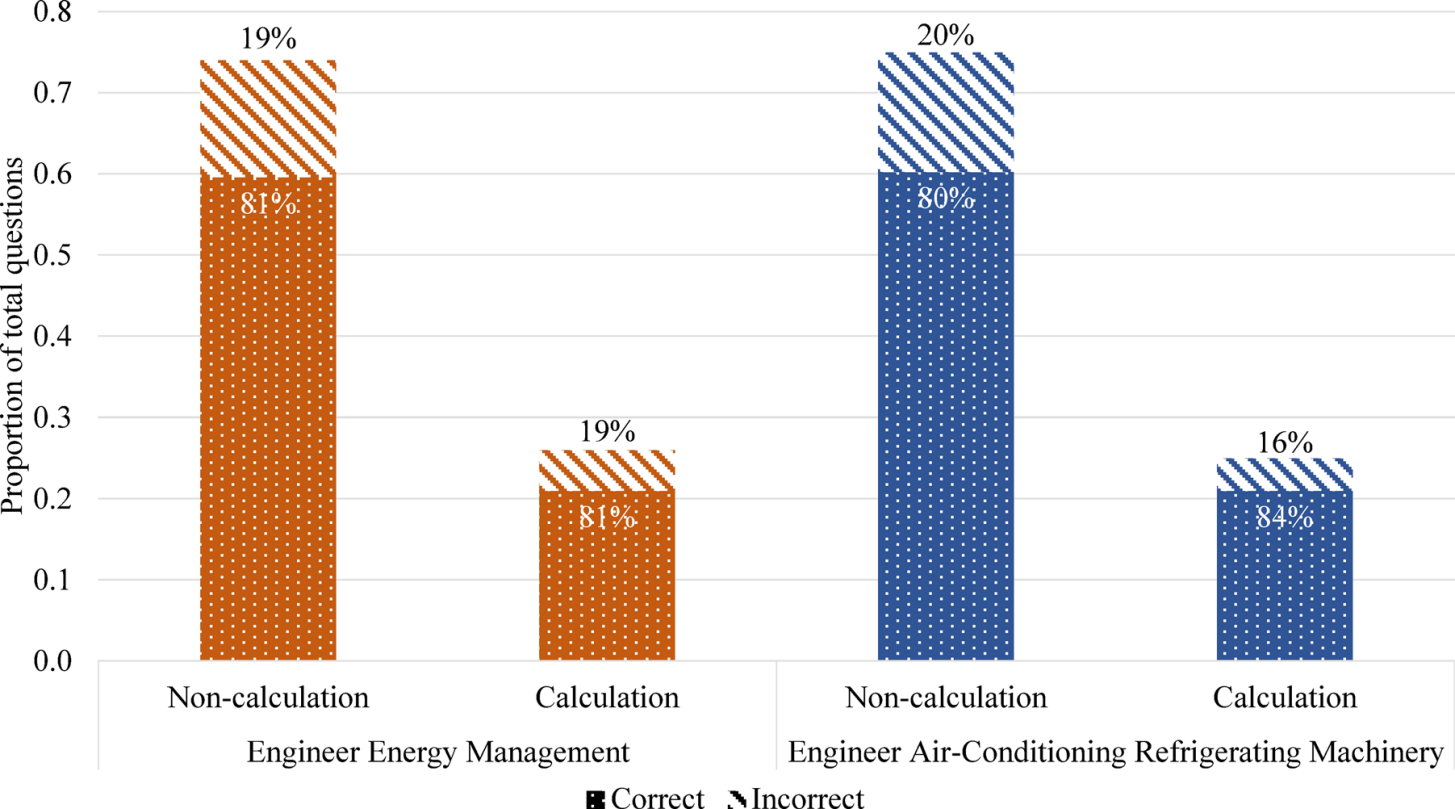
Ratio of problems by type and accuracy for each problem type.

The accuracy for each problem type is shown in Fig. 7. For the EEM exam, the average accuracy rate for non-calculation problems was 81%, while the average accuracy rate for calculation problems was also 81%. Similarly, in the EACRM exam, the average accuracy rate was 80% for non-calculation problems and 84% for calculation problems, showing minimal differences between the two types. Although the number of non-calculation problems was, on average, three times higher than that of calculation problems in both exams, the accuracy of responses did not vary based on problem type. Some studies have reported that GPT is vulnerable to exact calculations^31,32^; however, this study did not find such evidence. In fact, the findings of this study align with OpenAI’s technical report, which stated that GPT performed equally well in reading and math on the SAT, ranking in the top 7% and 11%, respectively^20^.

#### Consistency

**Fig. 8:**
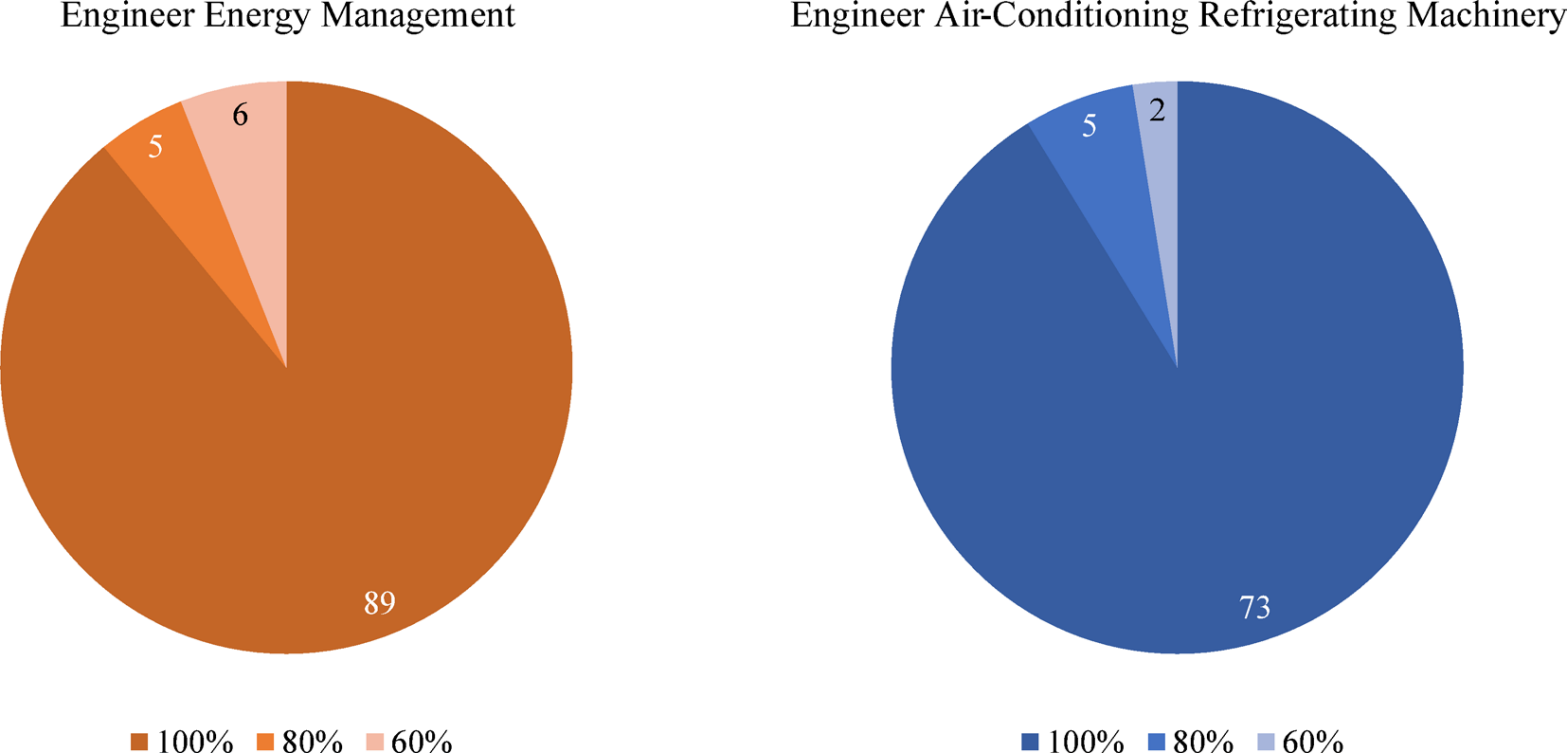
Consistency distribution of exam questions.

The consistency distribution of exam questions is shown in Fig. 8. In the EEM exam, the average consistency across five attempts was 97%. A total of 89 questions achieved 100% consistency, while only 11 questions recorded a consistency rate between 60% and 80%. In the EACRM exam, the consistency averaged 98%. A total of 73 questions achieved 100% consistency, while only 7 questions recorded a consistency rate between 60% and 80%. This high level of consistency is attributed to setting the temperature, a model hyperparameter, to 0.2, a very low value. Since the exam does not require creative responses, it is crucial to answer consistently based on existing knowledge, regardless of whether the answer is correct or incorrect. Therefore, achieving a consistency rate of over 97% across both exams validates the methodology of this study, confirming that setting the hyperparameter temperature to 0.2 is an appropriate approach.

#### Error distribution

The distribution of error types is summarized in Table 2. A total of 172 erroneous responses were identified across five test attempts, consisting of 48 reasoning errors (28%), 104 knowledge errors (60%), and 20 context errors (12%). Among these, knowledge errors accounted for the largest proportion.

**Table 2:** Number of error responses by type across EEM and EACRM exams.

	Total errors	Reasoning error	Knowledge error	Context error
EEM	97	32 (33%)	60 (62%)	5 (5%)
EARM	75	16 (21%)	44 (59%)	15 (20%)
**Total**	**172**	**48 (28%)**	**104 (60%)**	**20 (12%)**

Reasoning errors were observed only in calculation problems. These errors included incorrect equation setup, computational mistakes, and unit conversion errors, indicating GPT-4o’s limitations in logical reasoning and quantitative processing when solving formula-based questions. Knowledge errors occurred exclusively in non-calculation problems. These errors resulted from inaccurate recall of factual knowledge, such as engineering principles or legal information, and suggest that GPT-4o may lack sufficiently refined internal knowledge in certain domain-specific contexts. Context errors were found only in problems requiring figure interpretation, highlighting GPT-4o’s difficulty in visual reasoning when understanding the figure is essential to solving the problem.

Based on the error distribution analysis, this study selected representative cases from each error category that warranted further examination, and explored them in greater depth in the subsequent section, *Limitation of problem-solving*. Specifically, three subsections—*Lack of advanced reasoning ability*, *Difficulty in solving legal questions*, and *Weakness in interpreting figures*—were presented to qualitatively analyze key vulnerabilities in GPT-4o’s problem-solving performance.

### Qualitative evaluation

While the error distribution provided a quantitative overview of GPT-4o’s limitations, it does not fully capture how the model understands and responds to different problem types. To complement this analysis, we begin by examining representative examples of correct answers, focusing on both non-calculation and calculation problems.

#### Problem-solving ability for non-calculation problems

**Fig. 9:**
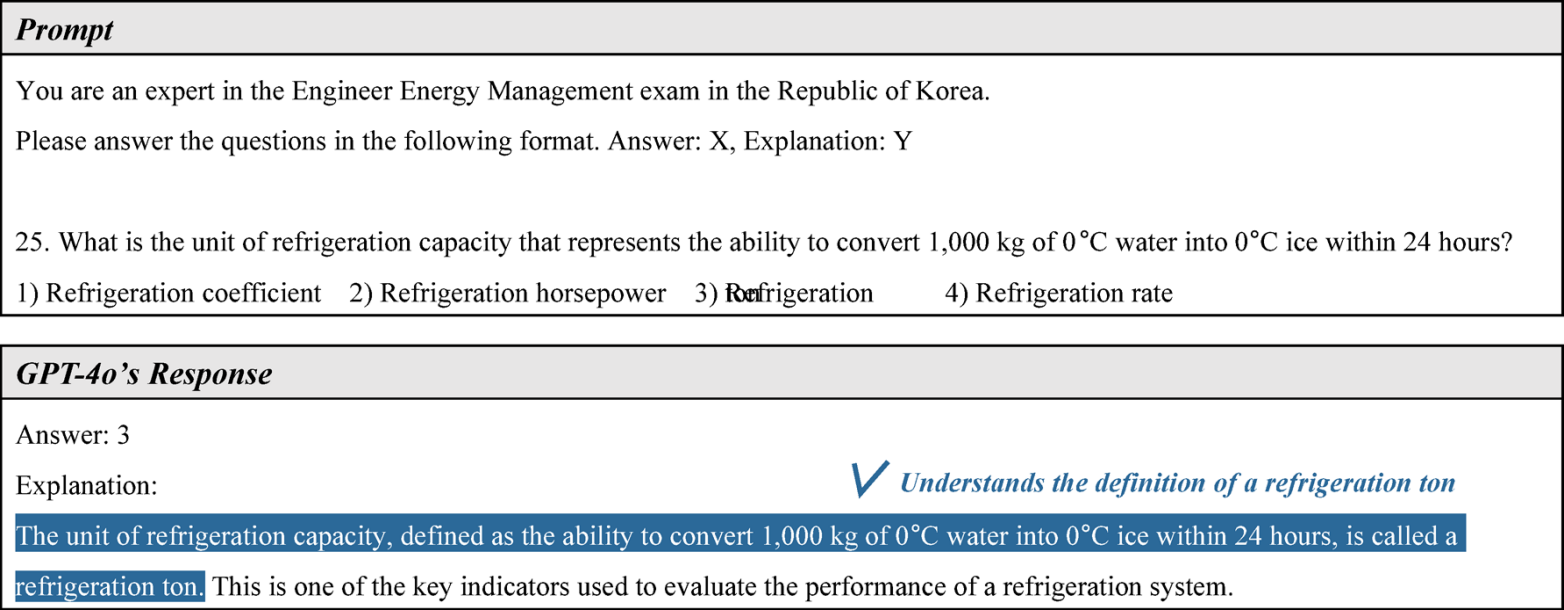
First example of non-calculation problem solving.

First, EEM exam question 25 and GPT-4o’s response are shown in Fig. 9. This is a warm-up question asking for the unit of refrigeration capacity. GPT correctly answered: “The unit of refrigeration capacity, defined as the ability to convert 1,000 kg of 0 °C water into 0 °C ice within 24 hours, is called a refrigeration ton.” This response demonstrates that GPT-4o understands fundamental terminology related to mechanical equipment.

**Fig. 10:**
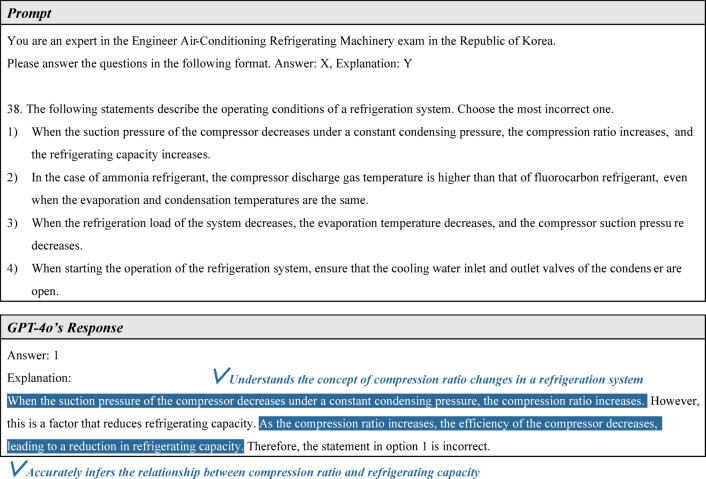
Second example of non-calculation problem solving.

Next, EACRM exam question 38 and GPT-4o’s response are shown in Fig. 10. This question pertains to the compression ratio of a refrigeration system. Examining GPT-4o’s answer, it is evident that the model is familiar with the fundamental concept of compression ratio variation: “When the suction pressure of the compressor decreases under a constant condensing pressure, the compression ratio increases.” What is particularly noteworthy is that GPT-4o accurately inferred the relationship between the compression ratio and refrigeration capacity, stating: “As the compression ratio increases, the efficiency of the compressor decreases, leading to a reduction in refrigeration capacity.” This response indicates that GPT-4o not only recalls fundamental knowledge related to mechanical equipment but also has the capability to infer relationships between different concepts.

#### Problem-solving ability for calculation problems

**Fig. 11:**
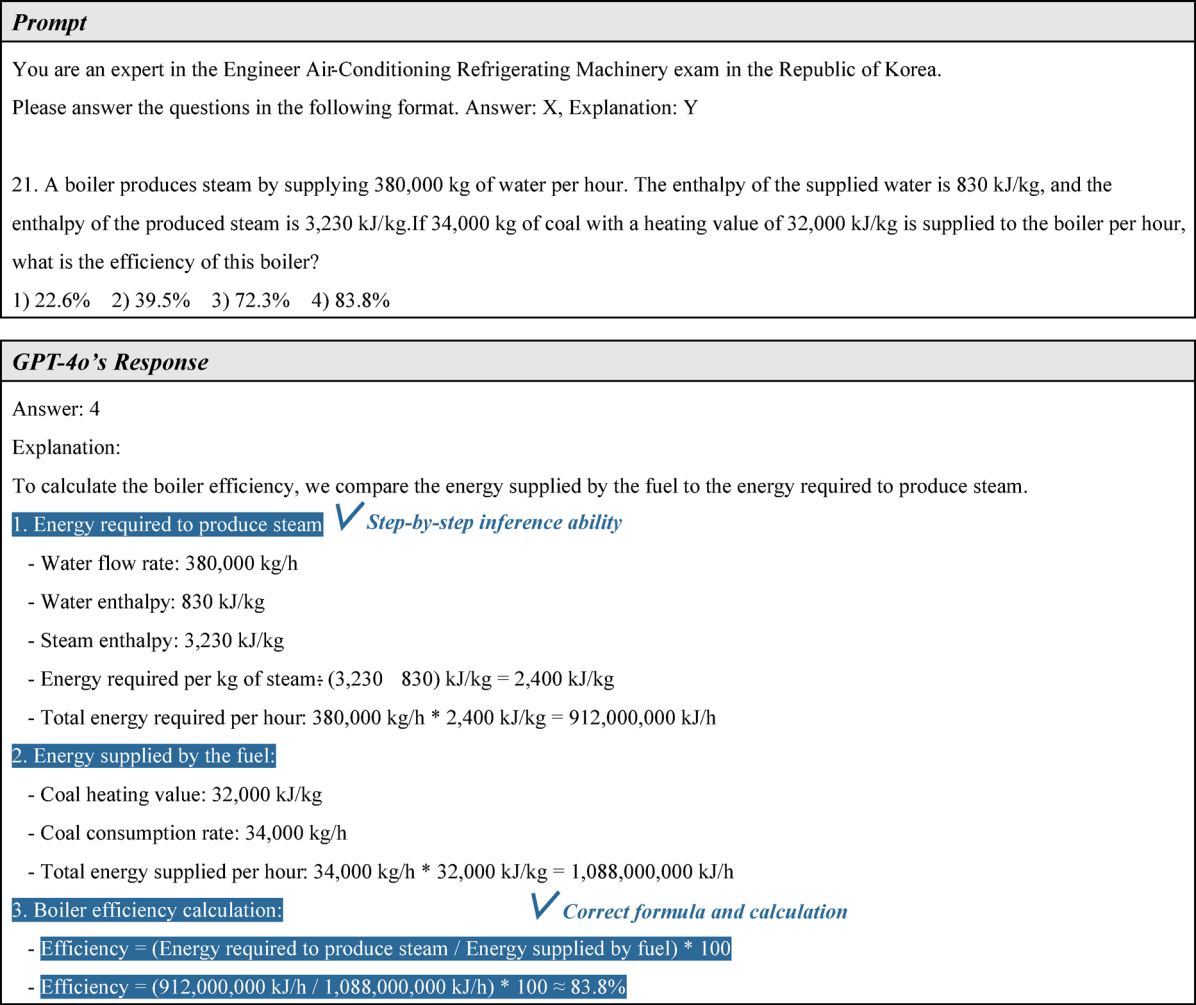
First example of calculation problem solving.

The following is an analysis of GPT-4o’s performance on a calculation problem. In the exam, calculation problems are based on the theory of mechanical equipment and generally require more inference steps than non-calculation problems. Figure 11 presents question 21 from the EACRM exam along with GPT-4o’s response. This problem is particularly challenging, as it requires calculating boiler efficiency using various given data points. Overall, GPT-4o provided a well-structured and accurate solution. It first calculated the energy of the steam produced by the boiler, then determined the energy of the fuel supplied to the boiler, and finally computed the efficiency using these values. Additionally, the calculations, including unit conversions, were performed correctly, leading to the correct final answer. These results demonstrate that GPT-4o possesses a high-level, step-by-step inference ability based on its knowledge of mechanical equipment and is capable of performing complex calculations with precision.

### Limitations of problem-solving

As described in the error distribution analysis, GPT-4o’s incorrect responses can be categorized into three types: reasoning errors, knowledge errors, and context errors. To better understand the nature of these mistakes, this section presents a qualitative analysis of representative failure cases. Specifically, it highlights three key limitations that correspond to the major error types identified earlier: lack of advanced reasoning ability, difficulty in solving legal questions, and weakness in interpreting scientific figures. These analyses provide insight into GPT-4o’s current boundaries and suggest areas for future improvement.

#### Lack of advanced reasoning ability

While GPT-4o exhibited strong reasoning ability based on its knowledge of mechanical equipment maintenance, it occasionally made inferences based on incorrect background knowledge or provided incorrect answers due to logical flaws. In this study, such errors are classified as incorrect answers resulting from a lack of advanced reasoning ability.

**Fig. 12:**
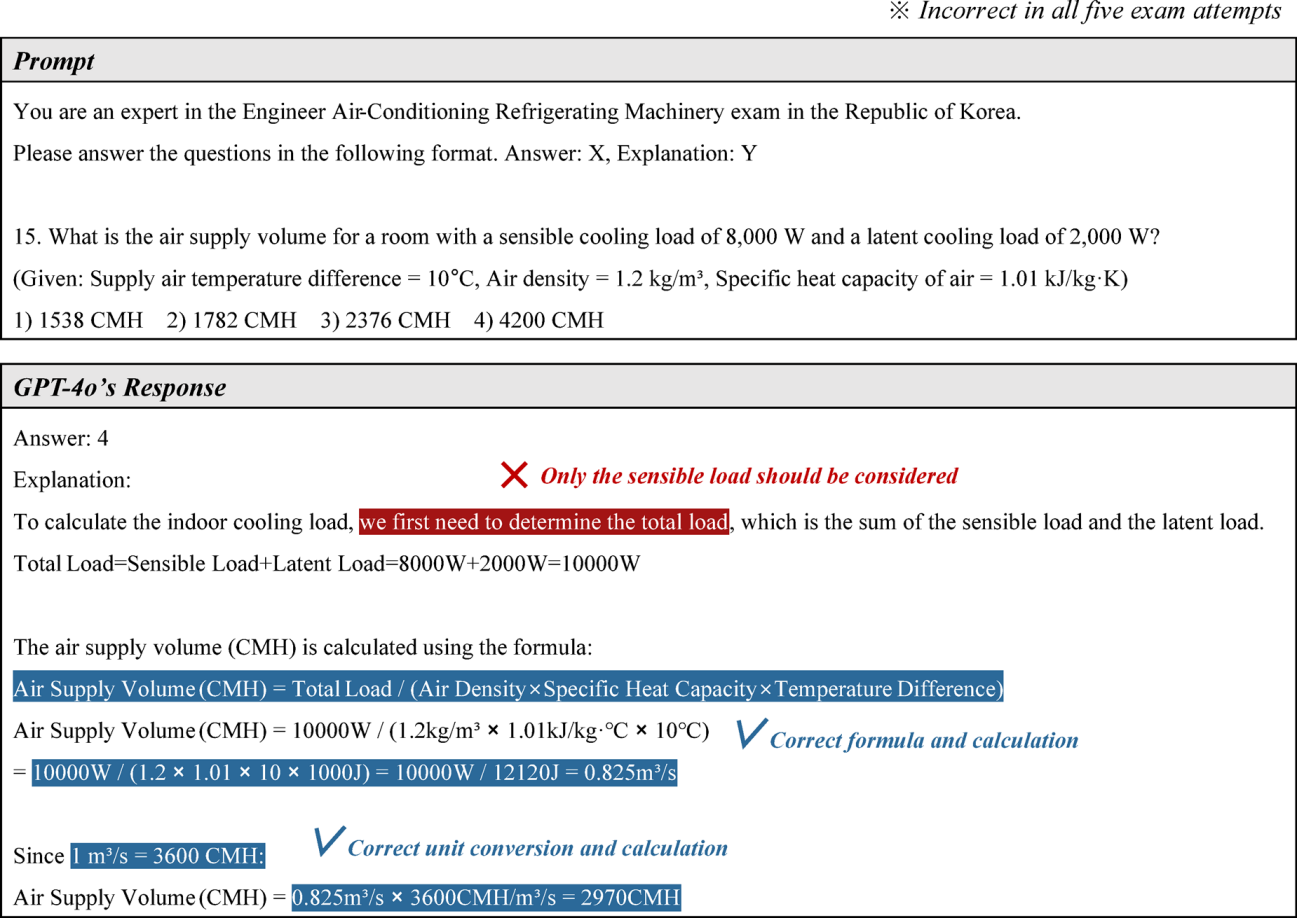
An example of incorrect answers resulting from a lack of advanced reasoning ability.

A representative example can be found in question 15 of the EACRM exam, as shown in Fig. 12. This problem requires calculating the appropriate airflow rate for a room based on given information. It is particularly important because it assesses fundamental knowledge related to equipment capacity design. However, the problem contains certain traps. First, although only sensible heat is typically considered when calculating airflow, the problem also provides latent heat as additional information. Second, even if latent heat were to be considered, the problem does not provide enthalpy values, which are necessary for calculation. GPT-4o fell into this trap and answered the problem incorrectly in all five attempts. As indicated in the first line of its response, the error occurred in the cooling load calculation, where it mistakenly included both sensible heat and latent heat in the total load. Although its subsequent airflow calculation formula, numerical computation, and unit conversion were correct, the final answer was incorrect.

Given GPT-4o’s limitations in advanced reasoning, an important question arises: is there a way to enhance its reasoning capabilities? Fortunately, models with improved reasoning abilities, such as OpenAI’s o1^33^, have recently emerged. Unlike traditional LLMs that generate answers based solely on learned data, o1 is designed to employ various problem-solving strategies, learn from mistakes, and demonstrate deeper reasoning ability. To evaluate these improvements, this study tested o1-preview (the version integrated into ChatGPT as of August 2024) on three questions that required advanced reasoning and on which GPT-4o failed all five attempts. The results showed that o1-preview achieved a 100% accuracy rate on these problems.

**Fig. 13:**
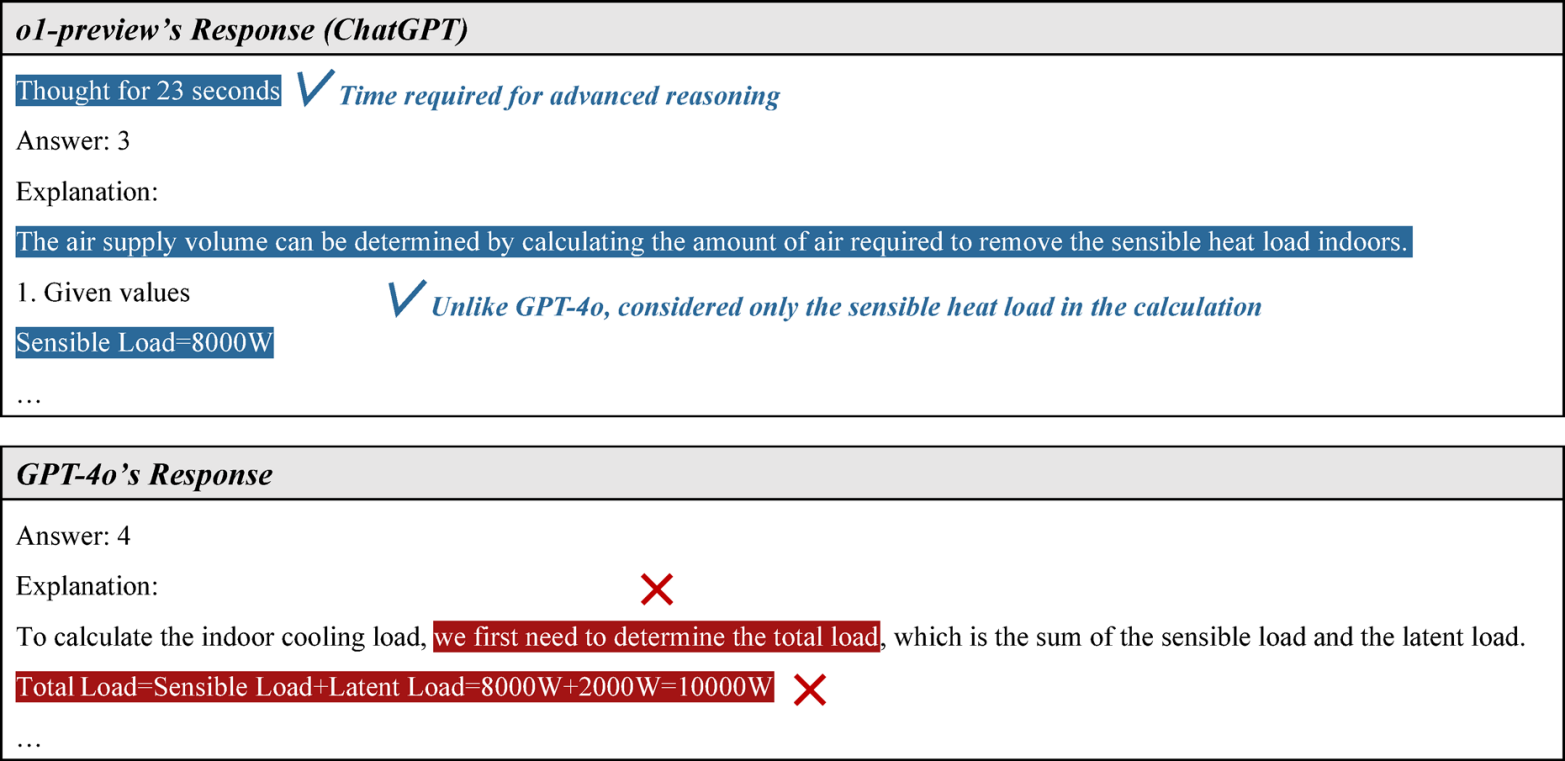
Comparison of o1-preview and GPT-4o responses.

As an example, Fig. 13 compares the responses of o1-preview and GPT-4o to question 15 of the EACRM exam. In this comparison, o1-preview provided an accurate answer by correctly considering only sensible heat when estimating the load, whereas GPT-4o mistakenly included latent heat, leading to an incorrect response. This suggests that reasoning models may be able to mitigate certain reasoning errors observed in earlier versions. These findings indicate that LLMs could become increasingly useful in the field of mechanical equipment maintenance as reasoning-enhanced models continue to be actively developed.

In addition to o1-preview, models such as Google’s Gemini 2.0 Flash Thinking and DeepSeek’s DeepSeek-R1 have been designed to enhance logical inference and problem-solving accuracy. As such models continue to advance, they are expected to play a more significant role in complex technical fields, including mechanical equipment maintenance. While these models show strong potential, their current cost may limit access for some users—a factor worth considering as the technology continues to evolve. Moreover, as reasoning models become more sophisticated, they may also produce more convincing but incorrect outputs^34^suggesting that such tools, while powerful, should not be uncritically trusted.

#### Difficulty in solving legal questions

Another limitation of GPT-4o is its difficulty in solving legal questions. Legal questions differ significantly from general knowledge questions in two key ways. First, they are not based on broad general knowledge but rather on highly specific regulations of a particular country, making them domain-specific knowledge. Second, legal regulations are not fixed but are subject to amendments over time, making them changeable knowledge. These characteristics can negatively impact the response performance of LLMs. Domain-specific data, which is relatively limited in quantity, has minimal influence on LLM training, and once an LLM is trained on past data, it does not update with new information until the next training cycle. These limitations stem from the way current LLMs are trained—based on static datasets with no built-in mechanisms for real-time updating. As a result, they often struggle in domains like law where accuracy depends on timely and jurisdiction-specific knowledge.

**Fig. 14:**
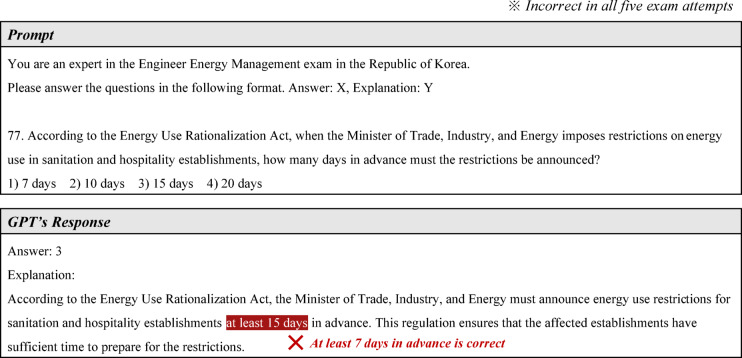
Example of an incorrect answer to a legal question.

Due to these factors, GPT-4o showed a low average accuracy rate of 68% for legal questions across both exams, which is significantly below the overall average accuracy rate of approximately 80%. A representative example is question 77 from the EEM exam, shown in Fig. 14. This problem is a legal question asking about the advance notice period required when restricting energy use under South Korea’s “Energy Use Rationalization Act.” It is a straightforward question that does not require calculations or theoretical reasoning but simply knowledge of the relevant law. Despite this, GPT-4o provided completely irrelevant answers in all five attempts.

Given this limitation, is there a way to improve GPT-4o’s legal question-solving ability? Fortunately, a technology called Retrieval-Augmented Generation (RAG) has recently gained attention. RAG enables LLMs to reference an external knowledge base beyond their training data before generating a response, allowing them to adapt more flexibly to domain-specific or newly updated knowledge (e.g., when laws change)^35^. This approach is also significantly more cost-effective than fine-tuning a model with domain-specific data^36^.

**Fig. 15:**
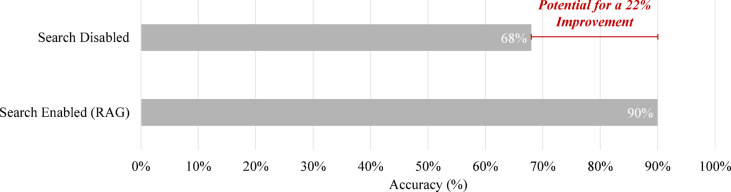
Results of search-based legal question solving across both exams.

There are various methods for retrieving new data in RAG, such as referencing documents (e.g., PDFs) or utilizing Internet searches. In this study, GPT-4o was re-tested in ChatGPT with search-based retrieval enabled for legal questions. When search was allowed, ChatGPT retrieved information from the correct source (law.go.kr, Ministry of Government Legislation) and produced the correct response. Overall, accuracy improved across all 16 legal questions from the two exams. When ChatGPT was asked to solve all 16 questions five times with search enabled, it achieved a 90% accuracy rate—a 22% improvement compared to when search was not allowed (Fig. 15). If search had been enabled for all questions, the average overall score across both exams would have exceeded 85 points, demonstrating the potential benefits of integrating RAG into LLM-based problem-solving.

These findings suggest that incorporating real-time retrieval methods into LLMs could significantly improve their ability to answer domain-specific questions accurately, not only in the legal field but also in areas such as building engineering^37,38^. As RAG continues to gain traction, it offers a promising direction for enhancing LLM performance in domains where information changes frequently or requires high specificity. However, its reliance on external search APIs or platforms also presents practical considerations—such as long-term service stability and system maintenance—that should not be overlooked in real-world deployments.

#### Weakness in interpreting figures

The third limitation observed in this study is GPT-4o’s difficulty in interpreting figures. In the EEM exam, it correctly answered only one out of three figure interpretation questions, while in the EACRM exam, it correctly answered only one out of five. Although there were only eight figure interpretation questions across both exams, this issue cannot be overlooked, considering that figure analysis—such as interpreting engineering schematics and psychrometric charts—is essential in the field of mechanical engineering.

**Fig. 16:**
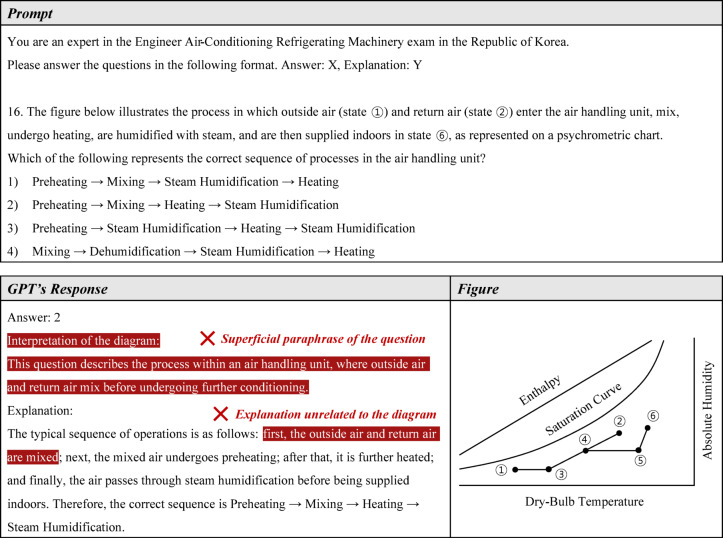
Example of weakness in figure interpretation.

A representative example is shown in Fig. 16, which presents question 16 from the EACRM exam, a problem that evaluates the ability to interpret a psychrometric chart. Understanding the psychrometric chart is fundamental knowledge for air conditioning design. Interestingly, GPT-4o selected the correct answer in all five attempts, yet its detailed explanation was completely incorrect. The first line of its response described the figure, but this was merely a superficial paraphrase of the question rather than an actual interpretation. Moreover, instead of analyzing the figure itself, GPT-4o provided general knowledge unrelated to the specific visual data. The figure illustrates a process in which outside air enters the air handling unit, is preheated, and then mixes with the return air. However, GPT-4o’s response incorrectly stated that the outside air and return air mix directly, demonstrating that it did not fully understand the figure.

GPT-4o’s ability to solve image-based questions differs from its ability to solve general text-based questions. To correctly answer an image-based question, GPT-4o must first interpret the image accurately. If an error occurs during this interpretation process, the model fails to leverage its reasoning ability, ultimately leading to incorrect answers. Therefore, failing to answer an image-based question correctly does not necessarily indicate a lack of knowledge in mechanical equipment. Given that GPT-4o demonstrated a strong understanding of mechanical concepts and reasoning ability in this study, its difficulty in answering image-based questions likely stems from a limitation in image interpretation rather than a lack of domain knowledge.

To enhance the image interpretation ability of LLMs, or more precisely, large multimodal models, it is essential to improve visual recognition performance. While deep learning-based visual recognition technology has made significant progress, there is still room for improvement in interpreting basic visual elements, such as lines and graphs. For example, recent studies have shown that GPT’s straight-line detection performance is suboptimal^39^that it relies more on background knowledge than on a deep understanding of visual data^16,40^and that it struggles to accurately interpret graphs in specialized fields such as bioinformatics^41^ and kinematics^42^. These findings indicate that the visual recognition capabilities of large language models still require further development and that more time will be needed before scientific figure interpretation can be reliably applied in industrial and engineering fields that utilize LLMs.

## Conclusion

This study was conducted to evaluate the applicability of large language models in the field of mechanical equipment maintenance in South Korea. To achieve this, GPT-4o was tested on the written exams for Engineer Energy Management (EEM) and Engineer Air-Conditioning Refrigerating Machinery (EACRM), both of which are nationally recognized qualifications required for mechanical equipment maintenance. The results were analyzed in detail, and the key findings are summarized as follows.

- First, in the quantitative performance evaluation, GPT-4o achieved high scores, recording an average of 80.6 points (SD = 1.82) on the EEM exam and 81.25 points (SD = 0.88) on the EACRM exam. The pass rate for both exams was 100% across five attempts. The model demonstrated high accuracy in both non-calculation and calculation questions, with almost no difference in performance between the two types. Furthermore, by adjusting the hyperparameters, it was possible to maintain high consistency in its responses. An analysis of error distribution showed that the majority were knowledge errors, followed by reasoning and context errors, providing insight into specific patterns of failure despite the overall high scores.
- Second, in the qualitative performance evaluation, GPT-4o exhibited an ability to infer relationships between knowledge, going beyond simple memorization in non-calculation questions related to mechanical equipment. Additionally, for calculation questions, it demonstrated advanced step-by-step reasoning based on mechanical equipment knowledge. The model was also capable of handling complex calculations and unit conversions with a high degree of accuracy.
- Despite these strengths, three major limitations were identified, which align with the error types revealed in the quantitative analysis: (1) lack of advanced reasoning ability, (2) difficulty in solving legal questions, and (3) weakness in interpreting figures. The limitation in advanced reasoning may be alleviated through the use of recently developed models with enhanced reasoning capabilities. The difficulty in solving legal questions stems from their domain-specific and frequently changing nature, but this issue can be partially mitigated using techniques such as RAG. Given the rapid advancements in reasoning models and RAG, there is clear potential to address these limitations, although the extent and timing of such improvements remain uncertain. However, weaknesses in figure interpretation are directly dependent on the visual recognition capabilities of models. Therefore, the effective application of LLMs in industrial fields may depend on future improvements in visual recognition technologies are achieved.

The findings of this study serve as a foundation for further discussions on how LLMs can be effectively integrated into the mechanical equipment industry. Future research should explore methodologies and practical applications of these models in software solutions such as Building Automation Systems (BAS), Building Energy Management Systems (BEMS), and Energy Management Information Systems (EMIS), which are widely used in mechanical equipment maintenance. Through such applications, LLMs could help address the manpower shortage in mechanical equipment maintenance, which was the primary motivation for this study. For instance, an LLM could be integrated into a BEMS platform to assist field engineers by interpreting fault alarms and offering preliminary diagnostic suggestions drawn from equipment manuals or historical data. In such use cases, the model would act as a collaborative assistant, supporting human technicians in real time while leaving final decisions and actions to experienced personnel.

Despite its meaningful contributions, this study has several limitations. First, given the rapid pace of language model development, the findings may not fully reflect the performance of the latest models. Continuous follow-up research is therefore needed to evaluate newer models as they emerge. Second, the study evaluated GPT-4o’s capabilities using static, exam-based metrics, which do not fully capture the complexity of real-world mechanical maintenance tasks. Certification exams assess domain knowledge in structured, text-based formats, whereas field operations involve unstructured problems, physical manipulation of equipment, and real-time decision-making. While the exam results indicate that GPT-4o possesses foundational engineering knowledge, this does not guarantee practical effectiveness in dynamic environments. Future work should examine how LLMs can be combined with sensors, control systems, or middleware platforms to enable real-world task execution. Lastly, since the study focused on exams conducted in South Korea, the findings may not be fully generalizable to other regions. While the general engineering knowledge assessed in the exams is likely universal, further validation is needed to assess the model’s knowledge in diverse regulatory and operational contexts.

## Data Availability

The data that support the findings of this study will be made available from the corresponding author upon reasonable request.
